# Early-BYRD: alternative early pacing and defibrillation lead replacement avoiding venous puncture

**DOI:** 10.1186/s13019-018-0795-5

**Published:** 2018-10-03

**Authors:** Andreas Keyser, Simon Schopka, Carsten Jungbauer, Maik Foltan, Christof Schmid

**Affiliations:** 1 Department of Cardiothoracic Surgery, University Medical Center Regensburg, Franz-Josef-Strauss-Allee 11, 93053 Regensburg, Germany; 2 Department of Internal Medicine II/Cardiology, University Medical Center, Regensburg, Germany

**Keywords:** Pacemaker, Implantable cardioverter defibrillator, Lead exchange, Polypropylene sheath, Venous puncture

## Abstract

**Background:**

In cases of lead failure after implantation of pacemakers (PM) or implantable cardioverter defibrillators (ICD) early lead replacement may be challenging. Puncture of the subclavian vein bears possible complications such as pneumothorax, hematothorax, and damage of leads to be left in place. To avoid venous puncture PM or ICD leads were replaced using a flexible polypropylene sheath (Byrd-sheath).

**Method:**

From January 2010 through December 2017, 55 patients underwent early lead exchange avoiding venous puncture. Early lead exchange for this study was defined as a reintervention within 14 days after the initial lead implantation. The connector of the malfunctioning lead was cut off, and stabilized by a stiff stylet. After having cut off the plastic knob of the stylet, the lead was passed through the polypropylene sheath and the latter advanced into the subclavian vein with gentle rotational movements to gain access to the subclavian vein. After lead removal the polypropylene sheath was replaced by a peel away sheath a new lead inserted.

**Results:**

Overall, 23 defibrillation leads and 34 pacing leads (16 right atrial leads, 17 right ventricular leads, and 1 left ventricular lead) were successfully explanted. Access to the subclavian vein was uneventful, and blood loss minimal. Radiation exposure and fluoroscopy time were also negligible.

**Conclusion:**

The Byrd-sheath technique proved to be safe and successful in providing vein access within 2 weeks after initial lead implantation using the previously implanted lead and thus avoiding puncture of the subclavian vein.

## Background

Malfunction or dislodgement of pacemaker (PM) or implantable defibrillator/cardioverter (ICD) leads should be corrected as they may result in significant morbidity. However, lead replacement procedures may pose certain risks after implantation of PMs or ICDs. Access to the subclavian vein may cause bleeding at the puncture site, as well as a hematothorax or pneumothorax especially in patients with chronic obstructive pulmonary disease, frailty, or cachexia [1–4]. Furthermore, the leads remaining can be damaged. Anticoagulants or platelet inhibitors increase the risk of bleeding complications with accidental puncture of the adjacent subclavian artery [4].

The manuscript presents results using a modified previously described method of exchanging pacemaker or defibrillator/cardioverter leads maintaining the venous access, and eliminating the need of venous puncture (Byrd-sheath technique) [5, 6].

## Method

From January 2010 to December 2017, 55 patients underwent early lead exchange avoiding venous puncture. Early lead exchange for this study was defined as reintervention up to 14 days after the initial lead implantation. All data had been prospectively collected in the institutional database.

### Patient data

Demographic data included age, gender, underlying diseases, and anticoagulation as well as the necessary information with regard to the initial implantation procedure. Procedural data contained time of procedure, time to gain access to the attempted vein, sheaths used, and fluoroscopy time. The procedural success and blood loss during the procedure were documented as well as postoperative pocket hematoma and infection.

### Surgical procedure

**Fig. 1:**
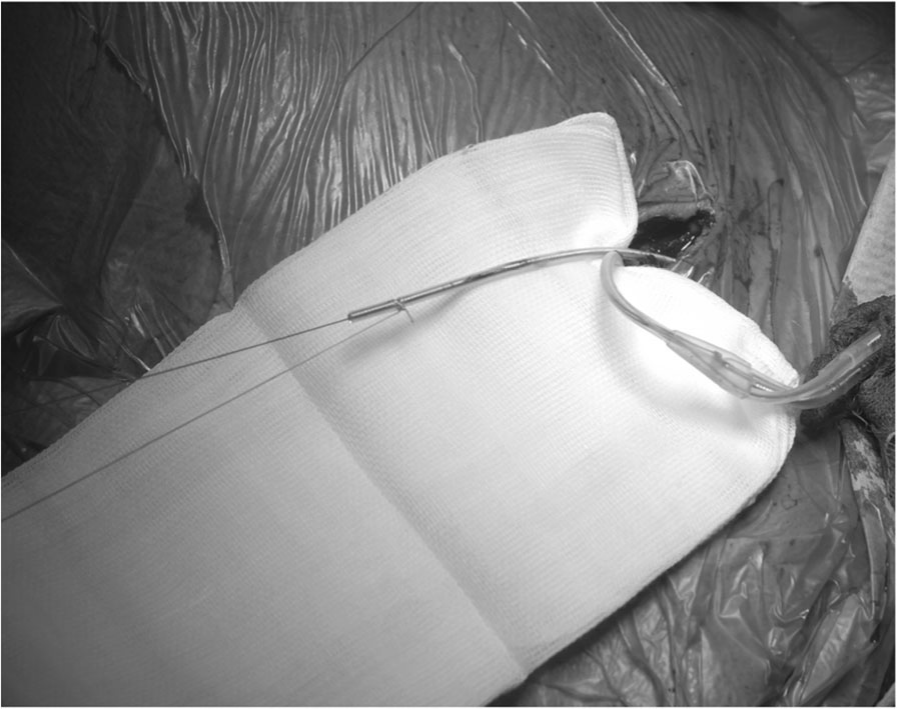
Preparation of the lead to be exchanged. Stiff stylet in lead to be exchanged, lead insulation fixed with suture (note ICD-lead being left in place)

**Fig. 2:**
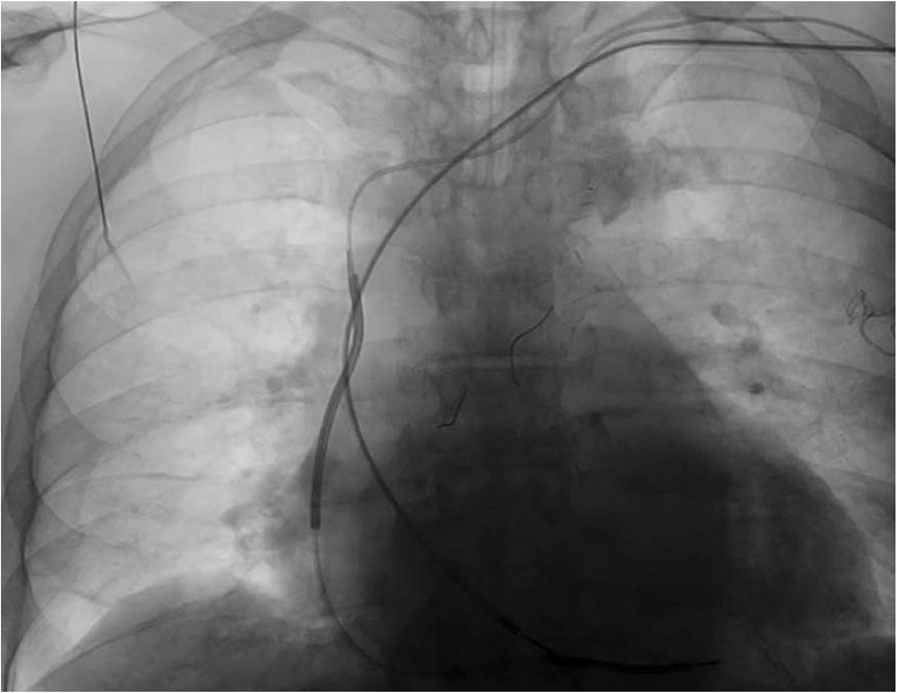
Radiofluoroscopy. Radioflouroscopic image of Byrd-sheath having been advanced over the lead (note the artrial lead had been loosened from active fixation for removal purose, the ICD lead remaining in place)

**Fig. 3:**
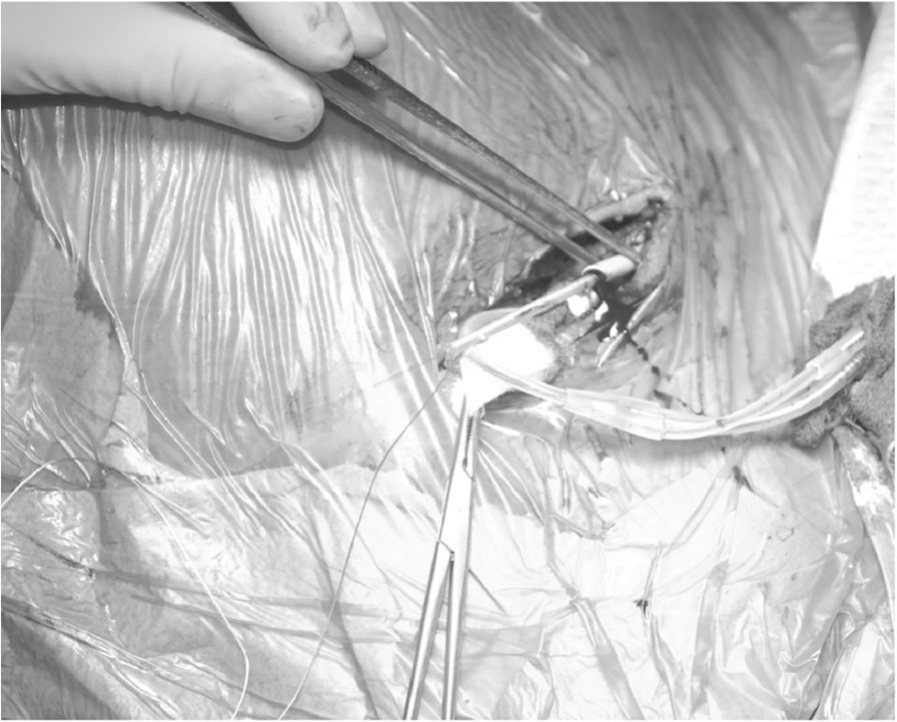
Access to vein. Byrd-sheath in subclavian vein (note blood dripping from sheath)

After having opened the generator pocket any sutures were removed from the lead to be replaced. Any other sutures (e.g. ligature of the cephalic vein) compromising the lead were also removed. Active fixations of leads were loosened. The connector of the lead was cut off and the suture sleeve removed. The lead was stabilized by a standard stiff stylet which was usually provided along with the new lead. The stiff stylet provided enough stiffness to allow advancement of a sheath over the lead. After the plastic knob of the stylet had been cut off, the insulation of the lead was fixed with a suture long enough to pass a flexible polypropylene sheath (Fig. 1). A polypropylene sheath of the size just to slide over the lead was chosen (Byrd Dilator Sheath Polypropylene, Cook Medical, Cook Inc., Bloomington, IN, USA). The sheath was gently advanced towards the subclavian vein with rotational movements using fluoroscopic control and assuring a gentle longitudinal movement without dislodging or distorting of the lead (Fig. 2). As soon as access to the vein was gained, the lead was removed. Access to the vein was indicated by dripping of blood from the polypropylene sheath (Fig. 3). Attention was drawn not to advance the sheath any further. A j-tipped guide wire was placed through the polypropylene sheath and a hemostatic peel-away introducer carefully advanced into the vein (SafeSheath®, Pressure Products Medical Supplies, Inc., Santa Barbara, USA or Prelude SNAP splittable sheath introducer, Merit Medical Systems Inc., Malvern, PA, USA).

### Statistical analysis

Statistical analysis was performed with Stata 10.1 SE for Windows (StataCorp., College Station, TX, USA). Continuous data were first tested for normality with the Shapiro–Wilk test. If normally distributed, these data are presented as means ± SD. Dichotomous data are expressed as numbers and percentages. The Mann–Whitney Rank sum test was used for non-normally distributed data. The tests were performed two-sided, and a *p*-value of < 0.05 was considered to be statistically significant.

## Results

Over a period of 8 years, 1946 patients with cardiac implantation of electrical devices were treated, thereof 525 revision procedures including all lead replacements. We detected 55 patients (35 male, 18 female) with early lead malfunction. Mean patient age was 67 ± 11 years (range 36 through 94 years). Twenty-four patients had a Phenprocumon or duel / triple platelet inhibition medication therapy.

Overall, 57 leads had to be replaced. Lead replacement was performed in 25 pacemaker systems (5 VVI, 20 DDD), and 30 ICD systems (14 VVI ICD, 10 DDD ICD, 6 CRT-D). Fifteen patients were referred to our institution for early lead exchange from other hospitals (27%). The indications for replacement were dislocation (*n* = 21), insufficient threshold (*n* = 22), and over−/undersensing (*n* = 14). Accordingly, 23 defibrillation leads, 16 right atrial leads, 17 right ventricular pace-sense leads, and 1 left ventricular lead had to be replaced. The mean dwell time of the leads after implantation was 3.6 ± 3.4 days (range 0 through 12 days). During the procedures, 40 leads in 34 patients remained in place. In 45 patients, the pacemaker or ICD was on the left side (82%), whereas 10 patients had a right-sided implantation (18%). A primary access via the cephalic vein was present in 15 patients (27%).

All procedures were successful. Mean time to gain access to the venous system was 2 ± 1 min. Blood loss was negligible in all cases. X-ray burden of the entire reintervention varied from 75.7 cGy*cm^2^ through 6740.7 cGy*cm^2^ (mean 1021.2 ± 1095.65 cGy*cm^2^) with time of fluoroscopy ranging from 0.24 min to 15.4 min (mean 3.28 ± 3.02 min). No postoperative pocket hematoma or infection developed. There were neither major nor minor complications.

We could not find a significant difference between patients with pacemaker or implantable cardioverter-defibrillator concerning the body mass index or age (*p* = 0.165; *p* = 0.479 respectively). Mean of ejection fraction was better in pacemaker patients compared to ICD patients (*p* = < 0.001). However, there was no difference when comparing pacemaker and ICD patients with respect to access time (*p* = 0.392), procedural time (*p* = 0.375), X-ray time (*p* = 0.819) and X-ray burden (*p* = 0.642).

Significant differences were also not found when comparing male with female patients with respect to ejection fraction (*p* = 0.189), as well as access and procedure time (*p* = 0.757; *p* = 0.785 respectively). Female patients though had a significant higher X-ray time (*p* = 0.026), but the X-ray burden appeared to be comparable and lacked statistical significance (*p* = 0.111).

An immediate lead exchange during the initial implant procedure occurred in 11 of the 55 patients. The procedure time was significantly higher in these patients (*p* = < 0.001). Likewise, the X-ray time and X-ray burden were significantly higher (*p* = < 0.001; *p* = 0.003 respectively).

## Discussion

Since Furman and Chardack introduced the first implantable pacemakers in the 1960s [7], the venous access has been improved by King [8] and Belott [9]. With increasing numbers of pacemakers and implantable cardioverter/defibrillators the quantity of lead malfunction steadily rises, and lead replacement remains a necessity and a challenge.

Puncture of the subclavian vein is common to gain access to the venous system in order replace malfunctioning leads. Complications associated with venous puncture are well described. Pneumothorax and hematothorax as major complications are rather infrequent, the incidence is described in 0.1–1.3% [1, 4]. Bleeding at the puncture pocket site as minor complication occurs in up to 2% [10, 11]. Most of the pocket hematomas are related to pocket formation rather than back from the venous system. Further, collateral lead damage is possible to other leads. This is exceedingly rare, though.

Major complications such as perforation of central veins and cardiac cavities have been described in causal connection with lead extraction or replacement [12]. Minor complications, such as pericardial effusion not requiring pericardiocentisis or surgical intervention, and hematothorax not requiring a chest tube, are linked to lead extraction [12]. Arm swelling or thrombosis of implant veins resulting in medical intervention, hematoma at the surgical site requiring reoperation for drainage, vascular repair near the implant site or venous entry site, blood transfusion related to blood loss during surgery, and pulmonary embolism not requiring surgical intervention are further adverse events classified as minor complications [12].

Using polypropylene sheaths to gain access to the venous system also bears the risk of bleeding, too, as the outer diameter is significantly larger than the lead body itself. Thorough surgery may well prevent bleeding back from the venous system even if a polypropylene sheath with is used. Sliding a properly sized peel-away introducer over the lead after it has been stabilized with a stiff stylet does not achieve the same results. The stiff stylet and the proximal end of the lead will have to be brought forward through the introducer cap in a retrograde manner. Especially when gaining the access to the subclavian vein, the rather soft tip of the peel-away introducer tends to be damaged by surrounding tissue. The damaged tip of the peel-away introducer may inhibit further advancement or even serious injury to the wall of the subclavian vein.

According to the analysis of Sant’Anna et al. we assume the proposed procedure safer, as we observed no bleeding complication with respect to our patients, as half of them were treated with oral anticoagulation or dual / triple platelet inhibition [13]. Neither bleeding from the puncture site nor bleeding as result of puncture of the axillary artery occurs when lead exchange is performed avoiding puncture techniques.

Air embolism is a rare but possible complication when using the polypropylene sheath technique. This complication did not occur and is avoided by keeping the patient in a slight Trendelenburg position during the procedure.

For primary venous access a retained guide wire technique is described by Byrd [14]. It may be helpful as a back-up method to avoid a second venous puncture in cases when the lead planted first has to be exchanged [15]. The guide wire is left in place in the sheath after the dilator is withdrawn, thus allowing the introduction of a second sheath to pass through a new pacing lead. However, this technique requires a larger intruding sheath and can be utilized only during the same implantation procedure when the first implanted lead is problematic. The guide wire itself may interfere with the lead placement. Having placed two guide wires with one introducer and using separate introducers for lead placement does not solve the interference and friction of multiple leads. In any case, these techniques may only be considered for primary implantation procedures.

A wire under the insulation technique to maintain venous access for the purpose of lead exchange has been previously described by Steinberg [16]. This technique may be employed in redo procedures. Yet, lead-on-lead interaction may render this approach challenging having two leads in place. Lead-on-lead interaction especially applies to silicone insulation layers. Even if the lead can be easily moved from the endocardium to the superior vena cava using direct traction, dislocation of the wire is possible with advancement of the lead. As the Byrd-technique indicates venous access as soon as blood is dripping out of the sheath, no further advancement of the sheath is required, thus simplifying he process. After the j-tipped guide-wire has been advanced, the access is secured. Once the access is assured, the situation will equal a normal lead implantation.

Leads with efficient passive fixation leads may be difficult to remove 4–6 months after implantation [14]. Fibrotic attachments develop between chronically implanted leads and venous, valvular and cardiac structures may pose obstacles to successful lead extraction [15]. The same applies to active fixation, as the fixation mechanism, the screw, may not be readily loosened. The reported technique is a modification of the described methods of Byrd and Bongiorni which allows overcoming these obstacles and may easily be used *during* or *early after* lead implantation, sacrificing the original lead(s) [5, 17].

This method may be employed within 1 year whenever lead malfunction occurs [14]. We focussed on early lead exchange ranging from intraoperatively to a maximum of 14 days since the initial implantation to eliminate adhesions possibly occurring within the first year of implant. The indication to sacrifice a lead with early malfunction is seen in bits of myocardium and/or small thrombi compromising the fixation mechanism. This applies to both active and passive fixation mechanisms of leads. Both fixation mechanisms may be impaired and unsatisfactory for proper and safe repositioning of a lead.

We could exchange all leads intended to be replaced leaving additional leads in place. Virtually any leads could be replaced, and any lead could be left in place without dislocation. It seems obvious, that obligate intraoperative lead exchanges proof to have the highest X-ray doses due to the difficulties in achieving a sufficient threshold and/or sensing during lead placement. As only a few seconds of additional time of fluoroscopy are required to gain access to the vein the additional X-ray burden remains negligible, especially when considering the entire procedural X-ray doses of lead exchanges [18]. None of the patients experienced complications due to the redo procedure.

As the implanted pacemaker devices are located in a layer under the pectoral fascia, the procedure of pacemaker lead replacement is well performed in local anaesthesia. We generally implant ICD devices in a sub muscular layer and accordingly approached defibrillator lead replacement in general anaesthesia, which is not mandatory. In any case, the technique of using a sheath for lead replacement itself does not oblige to a certain form of anaesthesia.

## Limitation

Our study has several limitations. It was designed as a retrospective study and conducted at one single medical centre. The number of reinterventions and referred patients for early lead exchange appears to be rather high. We interpret the referral of complex patients as a part of the duties of a department of cardiothoracic surgery. The study does not compare venous puncture with the described approach; there may be a selection bias. Experience in lead extraction may be of advantage.

## Conclusion

The technique of lead replacement using a flexible polypropylene sheath (Byrd-sheath) proved to be safe and successful in providing venous access using the previously implanted lead and thus avoiding puncture of a vein. The technique is simple and can be performed with standard operating room instruments. When clinically indicated, we advocate our method of replacing pacemaker or defibrillator/cardioverter leads in order to facilitate the procedure and minimize intraoperative complications.

## Abbreviations

CRT-D: cardiac resynchronization therapy defibrillator cardioverter
DDD ICD: duel chamber implantable cardioverter defibrillator
DDD: duel chamber pacemaker
ICD: implantable defibrillator cardioverter
PM: Pacemaker
VVI ICD: single chamber implantable cardioverter defibrillator
VVI: single chamber pacemaker

## References

[1] Eerola R, Kaukinen L, Kaukinen S (1985). Analysis of 13800 subclavian vein cathetherisations. Acta Anaesthesiol Scand.

[2] Byrd CL (1992). Safe introducer technique for pacemaker lead implantation. Pace.

[3] Haapaniemi L, Slatis P (1974). Supraclavivular cathetherisation of the superior vena cava. Acta Anaesthesiol Scand.

[4] Ruesch S, Walder B, Tramer MR (2002). Complications of central venous catheters: internal jugular versus subclavian access — a systematic review. Crit Care Med.

[5] Fearnot NE, Smith HJ, Goode LB, Byrd CL, Wilkoff BL, Sellers TD (1990). Intravsascular lead extraction using locking sylets, sheaths, and other techniques. PACE.

[6] Love CJ (2000). Current concepts in extraction of transvenous pacing and ICD leads. Cardiol Clin.

[7] Furman S, Schwedel JB (1959). An intracardiac pacemaker for stokes-Adams seizures. N Engl J Med.

[8] King SM, Arrington JO, Dalton ML (1968). Permanent transvenous cardiac pacing via the left cephalic vein. Ann Thorac Surg.

[9] Belott PH (1981). A variation to introducer technique for unlimited access to the subclavian vein. Pacing Clin Electrophysiol.

[10] Koh Y, Bingham NE, Law N, Le D, Mariani JA (2017). Cardiac implantable electronic device hematomas: risk factors and effect of prophylactic pressure bandaging. Pacing Clin Electrophysiol.

[11] Masiero S, Connolly SJ, Birnie D, Neuzner J, Hohnloser SH, Vinolas X, Kautzner J, O’Hara G, VanErven L, Gadler F, Wang J, Mabo P, Glikson M, Kutyifa V, Wright DJ, Essebag V, Healey JS (2017). Wound hematoma following defibrillator implantation: incidence and predictors in the Shockless implant evaluation (SIMPLE) trial. Europace.

[12] Maytin M, Epstein LM (2011). The challenges of tranvenous lead extraction. Heart.

[13] Sant’Anna RT, Leiria TL, Nascimento T, Sant’Anna JOM, Kalil RAK, Lima GG, Verma A, Healey JS, Birnie DH, Essebag V (2015). Meta-analysis of continuous Oral anticoagulants versus heparin bridging in patients undergoing CIED surgery: reappraisal after the BRUISE study. Pace.

[14] Byrd CL, Ellenbogen (2007). Managing device-related complications and transvenous lead extraction. Clinical cardiac pacing, defibrillation and resynchronization therapy.

[15] Wilkoff BL, Byrd CL, Love CJ, Hayes DL, Sellers TD, Schaerf R, Parsonnet V, Epstein LM, Sorrentino RA, Reiser C (1999). Pacemaker Lead extraction with the laser sheath: results of the pacing Lead extraction with the Excimer sheath (PLEXES) trial. J Am Coll Cardiol.

[16] Steinberg SD, Mayer DA, Tsapogas MJ, Wallack MK (2000). Pacemaker leads: a simple atraumatic method for replacing pacemaker electrodes. Ann Thorac Surg.

[17] Bongiorni Maria Grazia (2011). Personal Technique and Experience: The Pisa Approach. Transvenous Lead Extraction.

[18] Perisinakis K, Theocharopoulos N, Damilakis J, Manios E, Vardas P, Gourtsoyiannis N (2005). Fluoroscopically guided implantation of modern cardiac resynchronization devices. Radiation Burden to the Patient and Associated Risks. J Am Coll Cardiol.

